# The TVGH-NYCU Thal-Classifier: Development of a Machine-Learning Classifier for Differentiating Thalassemia and Non-Thalassemia Patients

**DOI:** 10.3390/diagnostics11091725

**Published:** 2021-09-20

**Authors:** Yi-Kai Fu, Hsueng-Mei Liu, Li-Hsuan Lee, Ying-Ju Chen, Sheng-Hsuan Chien, Jeong-Shi Lin, Wen-Chun Chen, Ming-Hsuan Cheng, Po-Heng Lin, Jheng-You Lai, Chyong-Mei Chen, Chun-Yu Liu

**Affiliations:** 1 School of Medicine, Yangming Campus, National Yang Ming Chiao Tung University, Taipei 112, Taiwan; yikaifu@gmail.com (Y.-K.F.); shulin2309@gmail.com (S.-H.C.); jslin@vghtpe.gov.tw (J.-S.L.); zmh09161721@gmail.com (M.-H.C.); cskplh0705@hotmail.com (P.-H.L.); 2 Department of Emergency Medicine, Far Eastern Memorial Hospital, New Taipei City 220, Taiwan; 3 Division of Transfusion Medicine, Department of Medicine, Taipei Veterans General Hospital, Taipei 112, Taiwan; liuhm@vghtpe.gov.tw (H.-M.L.); lhlee@vghtpe.gov.tw (L.-H.L.); yrchen@vghtpe.gov.tw (Y.-J.C.); wcchen16@vghtpe.gov.tw (W.-C.C.); 4 Institute of Clinical Medicine, Yangming Campus, National Yang Ming Chiao Tung University, Taipei 112, Taiwan; 5 Division of Hematology, Department of Medicine, Taipei Veterans General Hospital, Taipei 112, Taiwan; 6 Department of Ophthalmology, Taipei Veterans General Hospital, Taipei 112, Taiwan; 7 Department of Surgery, Kaohsiung Chang Gung Memorial Hospital, Kaohsiung 833, Taiwan; 8 Institute of Public Health, Yangming Campus, National Yang Ming Chiao Tung University, Taipei 112, Taiwan; yoio2580@gmail.com

**Keywords:** supportive vector machine, thalassemia, microcytic anemia, machine-learning

## Abstract

Thalassemia and iron deficiency are the most common etiologies for microcytic anemia and there are indices discriminating both from common laboratory simple automatic counters. In this study a new classifier for discriminating thalassemia and non-thalassemia microcytic anemia was generated via combination of exciting indices with machine-learning techniques. A total of 350 Taiwanese adult patients whose anemia diagnosis, complete blood cell counts, and hemoglobin gene profiles were retrospectively reviewed. Thirteen prior established indices were applied to current cohort and the sensitivity, specificity, positive and negative predictive values were calculated. A support vector machine (SVM) with Monte-Carlo cross-validation procedure was adopted to generate the classifier. The performance of our classifier was compared with original indices by calculating the average classification error rate and area under the curve (AUC) for the sampled datasets. The performance of this SVM model showed average AUC of 0.76 and average error rate of 0.26, which surpassed all other indices. In conclusion, we developed a convenient tool for primary-care physicians when deferential diagnosis contains thalassemia for the Taiwanese adult population. This approach needs to be validated in other studies or bigger database.

## 1. Introduction

Thalassemia and iron deficiency anemia (IDA) are the most common causes of microcystic anemia. Thalassemia is an autosomal recessive inherited genetic hemoglobinopathy with varying degrees of hypochromic microcytic anemia, depending on the genetic defects of alpha or beta globulin genes [1]. Traditionally, the prevalence of thalassemia in Mediterranean, Middle East, and Southeast Asian populations is much higher than in European and North American [2,3]. However, in this modern global society and the migration of human races, the prevalence of thalassemia may increase in other regions traditionally believed to have a low prevalence, whereas prevention and screening programs in endemic regions may reduce the number of affected individuals [4]. Among the Taiwanese population, the prevalence is 5% for alpha-thalassemia (αT) and 3.5% for beta-thalassemia (βT) [5]. In contrast, the prevalence of IDA in the general Taiwanese population has been reported as 0.2% in males and 2.1% in females, with the highest prevalence occurring in females of 30–50 years old [6]. Last but not least, AI is another common cause of microcytic anemia, especially in hospitalized and chronically ill patients. The causes of AI are associated with the impression of renal-produced erythropoietin by inflammatory cytokine and the decrease of iron availability for red blood cell development [7,8]. Differentiating thalassemia from IDA, AI, and other causes of microcytic anemia is clinically meaningful since the treatments for both are distinct [9,10].

Routine blood exams have shown high similarity between IDA and thalassemia, and complementary lab methods are needed [11]. Thalassemia is commonly diagnosed with the aid of hemoglobin electrophoresis, and increased HbA_2_ levels (>3.5%) often indicate presence of beta-thalassemia. However, diagnosis of thalassemia subtypes relies on genetic analysis for α- and β-globin genes [12]. In contrast, diagnosis of IDA is based on several serum biomarkers, including low ferritin, low transferrin saturation, raised total iron-binding capacity, raised red cell zinc protoporphyrin, or in combination [13]. AI is characterized by normocytic to microcytic mean corpuscular volume and elevated ferritin. It is necessary to exclude the possible coexisting thalassemia, IDA, blood loss, or medication effect, following which, the diagnosis of AI could be made [8]. All of the examinations require an additional outlay of time and expense. Besides, these assays may not be available in some thalassemia endemic areas, where health care resources are inadequate [14].

There are simple screening indices to differentiate thalassemia traits and IDA. They are usually derived from automated complete blood cell count parameters, including hemoglobin (Hb), red blood cell (RBC), mean corpuscular volume (MCV), mean corpuscular hemoglobin (MCH), mean corpuscular hemoglobin concentration (MCHC), and red blood cell distribution width (RDW) [15,16,17,18,19,20,21,22,23,24,25]. England and Fraser et al. introduced the first England and Fraser (E&F) index for discriminating IDA and βT trait (βTT) in 1973 [17]. Following this, the Mentzer index and Strivastava index both claimed to have better power than the E&F index in the absence of decreased RBC production or hemodilution [16,20]. In the next three decades, several indices aiming to discriminate βT minor and IDA have been proposed, including Shine and Lal (S&L), Ricerca, Green and King (G&K), RDW index, Sidah, Ehsani, mean density of Hb/liter of blood (MDHL) index, and mean cell hemoglobin density (MCHD) index [15,18,19,21,22,23,24]. Among these indices, the Mentzer index and RDW index have been widely used for their easy-calculating formula with fair sensitivity and specificity [26]. On the other hand, the Huber–Herklotz index (HH index) is one of the few indices that aimed at distinguishing αT trait (αTT) and IDA [25]. Each formula of the above-mentioned indices is listed in Supplementary Table S1, and the accuracy and performance of these indices vary in different population groups [27,28].

While all these indices may help differentiate thalassemia from IDA, the etiologies of anemia presenting in patients from hematology-specific outpatient clinics could be more complex, and multiple simultaneous causes of anemia, including AI, would exist [8]. Moreover, most of these indices either differentiate αTT from IDA or discriminate βTT from IDA. From a practical point of view, it is worth distinguishing the thalassemia and non-thalassemia for patients first followed by confirming genetic analysis. Otherwise, physicians could focus on the clinical evaluation and management of non-thalassemia etiologies by skipping unnecessary tests. For the purpose of helping the diagnosis process in outpatient settings, we combined existing indices with machine learning techniques to create a new formula for classifying thalassemia and non-thalassemia in Taiwanese adult patients who visit hematologists for anemia. This study developed and validated a new classifier that discriminates between IDA and thalassemia with improving performance, compared with former indices, using simple parameters provided by all automatic blood counters.

## 2. Materials and Methods

This study was conducted under the guidelines of the Helsinki Declaration and approved by the Institution Review Board (IRB) of the Taipei Veterans General Hospital (approval number 2021-05-025-CC). Because all identifying patient information was removed prior to analysis in this study, informed consent was waived upon approval by the IRB.

### 2.1. Baseline Demographics and Genomic Technique

We retrospectively reviewed the laboratory examination result of 350 patients with suspected thalassemia in Taipei Veterans General Hospital between January 2018 and January 2020. According to family history, clinical features, clinical features, or previous laboratory findings, these candidates were selected based on hematologist referral for thalassemia molecular tests. Patients under the age of 18 and who did not undergo globin gene mutation analysis were excluded. The baseline complete blood cell count, including white blood cell, Hb, RBC, MCV, MCH, MCHC, RDW, and platelet count were recorded, excluding any blood transfusion within three months. Based on the information, we computed 13 indices introduced previously, including Mentzer, RDWI, CRUISE, S&L, Srivastava, G&K, Sirdah, Ehsani, E&F, Ricerca, MDHL, MCHD, and HH according to the original published articles. Patients were then divided into one of three classes, αT, βT, and non-thalassemia, by genomic DNA analysis.

Genomic DNA was extracted from peripheral blood leukocytes using the Gentra Puregene Blood Kit (Qiagen, Hilden, Germany) according to the manufacturer’s instructions. The concentration of DNA was determined by NanoDrop Spectrophotometer ASP-2680 (ACTGene, USA). A multiplex gap-polymerase chain reaction (PCR) according to the methodology described by Arnold S.-C. Tan et al. [29] was used for detecting the five common α-thalassemia gene deletions in Taiwan, including ——SEA, ——FIL, ——THAI, —α3.7, and —α4.2. Non-deletional mutations in the α2-globin gene (HBA2), including the Hb Constant Spring, Hb Quong Sze, and Hb Westmead, which are the most common in the Chinese population, were detected by PCR/direct DNA sequencing. For identification of β-thalassemia mutations, whole β-globin genes (HBB) were PCR and direct sequencing by in-house primers. The PCR products were run on a 1% agarose gel using 1X TBE buffer, and lastly, the gel was visualized under ultraviolet (UV) light and the image was captured using Alphaimager system and software (Cell Biosciences, Santa Clara, CA, USA) [29].

The categorical data of the study patients were summarized as proportions and their difference was conducted by chi-square test. For continuous variables, descriptive results were summarized as mean ± standard derivation (SD) and their differences in mean were tested by the *t* test if normality assumption was satisfied or differences in median by Kruskall–Wallis method if normality assumption was violated.

### 2.2. General Concept of SVM

To classify thalassemia and non-thalassemia patients, we utilized the support-vector machine (SVM) with a radial kernel to perform classification. These machine learning tools with only a few tuning parameters have been applied to a wide range of prediction problems [30]. Since these indices were originally designed for distinguishing either αTT versus IDA or βTT versus IDA, directly building an SVM model performing binary classification as thalassemia and non-thalassemia would be easy to misclassify. For example, the patients with αTT may tend to be predicted as non-thalassemia due to some indices such as Mentzer or Sirdah since both mainly diagnose IDA and βTT. Moreover, the greatest proportion of the recruited patients are αTT (>50%) and the least are βTT (<15%), which would cause an imbalance problem. To avoid these problems, we propose a novel two-stage procedure to construct a classifier.

### 2.3. Detail Process of Two-Stage SVM Procedure

#### 2.3.1. Step 1. SVM for the Data with Three Classes, αTT, βTT, and Non-Thalassemia

- Randomly select two-thirds of the cohort as training data.
- Apply the “svm” and “tune” R package e1071 and use 10-fold cross validation to determine the best parameters of SVM and build the best classifier.
- Apply the principle of one-versus-one classification to classify each patient into one of αTT, βTT, and non-thalassemia.

#### 2.3.2. Step 2. Merge the Prediction Result of Being αTT or βTT into One Class, Named “Thalassemia”. The Classifier Performing Binary Classification, Thalassemia and Non-Thalassemia, Is Then Completed

**Figure 1:**
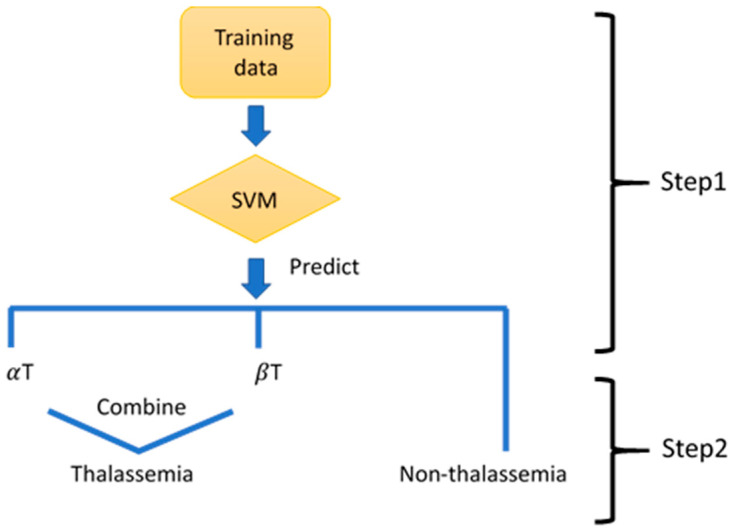
The diagram of building the classifier performing binary classification. Support vector machine (SVM), alpha-thalassemia (αT), beta-thalassemia (βT).

Due to the number of classes being more than two in step 1, we use the SVM with one-versus-one approach to predict the class of an individual [31]. The diagram of the proposed machine learning based classifier is shown in Figure 1.

To assess the performance of our proposed SVM approach for classifying thalassemia versus non-thalassemia, we randomly selected two-thirds of the cohort as training data for developing a classifier and the remaining one-third data as test data for assessing the performance of the classifier. The training data are randomly sampled from the original data without replacement. Based on the training data, the best machine is determined by using the aforementioned SVM approach. Given the best machine, the performance of the built machine is evaluated by calculating the classification error rate (CER) and the area under the curve (AUC) for the test data.

The currently developed indices, including Ricerca, Mentzer, RDWI, CRUISE, MDHL, S & L, Srivastava, G & K, Sirdah, Ehsani, E & F, MCHD, and HH, were applied for comparison. To this end, Monte-Carlo cross-validation procedure is employed, which could avoid over-optimism about prediction performance derived from the selected training data [32]. Hence, for all methods, we repeated the aforementioned process 1000 times to calculate and compare the average CER and AUC on test datasets.

All statistical analyses were performed using R statistical software, version 3.6.1. Two-tailed *p* values < 0.05 were considered to be statistically significant.

## 3. Results

### 3.1. Baseline Demographics

Among the 350 enrolled patients, 122 (34.8%) are of non-thalassemia, 179 (51.1%) of αT, and 49 (14%) of βT. The demographic characteristics is displayed in Table 1. The considered indices include Mentzer, RDWI, CRUISE, S&L, Srivastava, G&K, Sirdah, Ehsani, E&F, Ricerca, MDHL, MCHD, and HH. The distributions of these 13 indices are displayed via boxplots in Supplement Figure S1.

All characteristics and calculated indices are significantly different between αTT, βTT, and non-thalassemia groups except the MCHC and CRUISE indices. The sensitivity, specificity, positive prediction value (PPV), and negative prediction value (NPV) of the 13 indices to distinguish thalassemia and non-thalassemia are displayed in Table 2. The MCHD index has the highest sensitivity (99.12%) and NPV (81.82%) but with extremely low specificity (7.38%). The Huber–Herklotz index has the highest specificity (96.72%) with low sensitivity (9.73%). The highest PPV belongs to the RDW index (88.75%), with reasonable specificity (82.25%).

### 3.2. SVM Prediction Model

**Figure 2:**
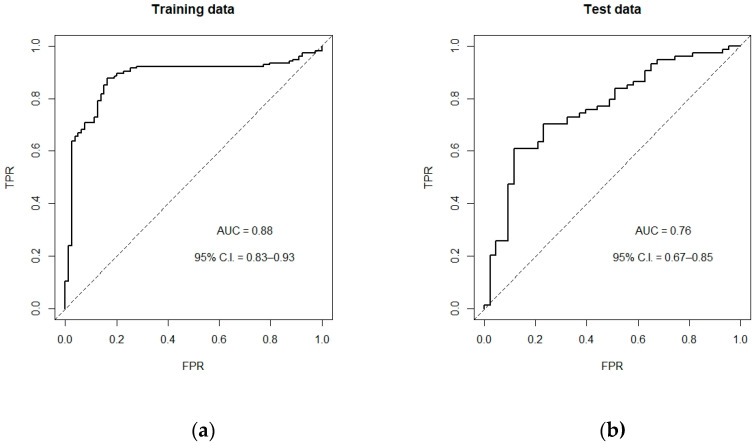
ROC curves of the proposed classifier. (**a**) The AUC and 95% C.I. based on the training data; (**b**) The AUC and 95% C.I. based on the test data.

In our SVM prediction model, 13 indices were included. Table 3 shows the 1000 average CERs (with standard deviation (SD)) and AUCs (with average 95% confidence intervals (C.I.)) of proposed model compared with all other 13 original indices. It can be seen that the proposed SVM prediction model owned the lowest error rate (0.26 with SD = 0.04) and the largest AUC value (0.76 with 95% C.I. = 0.69–0.86). Performance AUC samples using one randomly training dataset are showed in Figure 2, in which the AUC is 0.76 in the final testing data.

## 4. Discussion

It is crucial to differentiate the thalassemia trait, both alpha and beta, from non-thalassemia anemic diseases, most of the time, IDA. The complete diagnosis process required significant expense and time [12]. Thus, simple and stratified discrimination between these two groups could help in primary care facilities, especially where healthcare resources are inadequate [33]. We tried to use these existing indices to achieve our goal: classifying a patient to belonging to either the thalassemia or non-thalassemia groups. Unfortunately, none of these indices could do a great job. The commonly used RDW index had the best specificity and PPV in the current cohort. However, the unsatisfied sensitivity and NPV increased the prediction error rate up to 46%. Meanwhile, the MCHD index had the best sensitivity and NPV, but the 7.38% specificity makes it impossible to be a good predictor (Table 1 and Table 2). Indices developed in the past were mostly designed for distinguishing βTT and IDA; only the HH index is for αTT. We supposed that could be the main reason why individual indices cannot perform well in the prediction between the “thalassemia” and “non-thalassemia groups”. Once there were both αTT and βTT in the group of thalassemia, the indices designed for βTT and IDA performed poorly due to the presence of αTT and the same situation occurred when using the HH index.

When it comes to classification problems, support vector machine (SVM), a supervised machine learning technique, has its strengths. Between the two classes we wanted to distinguish, the SVM algorithm created a complex decision boundary. Using a radial kernel function in SVM, the algorithm mapped our data into a higher dimensional feature and created a hyperplane with the most significant margin and longest possible distance to the sample on each side. The larger the margin, the higher the accuracy could be [30]. In our study, the average performance of the SVM model had an AUC of 0.76 and an error rate of 0.26, which surpassed all other indices.

In addition, this study showed the strength and potential of using SVM as a possible solution to diagnose other diseases that have some original indices, but none of them are perfect.

All patients in our study were classified as thalassemia and non-thalassemia groups base on thalassemia DNA analysis. In this case, there was a possibility that there were thalassemia patients superimposed with IDA or AI. Because of the limitation of retrospective data collection, not all patients had available iron profiles. There were 171 patients whose iron profiles were available (110 in the thalassemia group, 61 in the non-thalassemia group). Among these 171 iron-profile-available patients, 39 patients may be considered IDA according to their iron profiles, and 24 of them had both IDA and thalassemia (21.8% among iron-profile-available thalassemia patients), whereas 15 of them belong to the non-thalassemia group (24.5% among iron-profile-available non-thalassemia patients). A recent report on the Taiwanese population showed that among 661 cases with thalassemia minors, 202 cases (31%) also had iron deficiencies [34]. 

Similarly, other causes of anemia, such as AI, may coexist with thalassemia. Nevertheless, this machine-learning-based algorithm was designated to reinforce the discrimination of thalassemia from non-thalassemia patients, whatever the other etiologies of anemia, and thus the need for further DNA examination. If non-thalassemia is confirmed, efforts should be made to seek other causes of anemia.

The study designs of previous studies of the 13 indices mentioned above all had strict inclusion and exclusion criteria. The majority of them only included βTT and IDA patients [15,16,17,20,21,22,24]; some of them even excluded those who had both βTT and IDA [19]. These criteria caused the index they created to have good performance in this specific patient group. However, when it comes to real clinical situations, where anemia patients might have multiple anemic causes, these indices became inaccurate and difficult to apply. In contrast, we only exclude patients under 18 years of age. The diversity of the anemic causes in the non-thalassemia group and the superimposing condition of thalassemia and IDA, AI, or other cause of anemia, made the training data more similar to clinical conditions. As a result, we firmly believe that our machine can have high application value for primary care physicians.

This is the first study using machine learning techniques to develop a thalassemia prediction model in the Taiwan adult population. Every case we collected in our study underwent DNA analysis for globin mutations. This analysis is the golden standard in current thalassemia diagnosis, which gave us a solid base and confidence. We retrieved data from 350 cases that underwent DNA analysis, the largest number in Taiwan among the research in thalassemia diagnosis models. To avoid the randomness derived from the selected training data, we repeated the aforementioned process 1000 times to calculate the average classification error rate. This process made the prediction ability of our model more convincing. We also transformed our model into website-based application, (available online: https://leader.doctorkeeps.com, accessed on 15 September 2021), where physicians can input the data of their patients who was suspected to have thalassemia. We also display the related indices which physicians can employ for their consideration. Physicians can reference the result and arrange the further appropriate examination of the patient. This online calculator could also be a potential tool for further data collection. 

Nevertheless, we can transform the online calculator and add a documentation function to record all typed data. Further real-life validation can be done with its help. We would like to perform a prospective validation study in a multi-center, large group of patients. The study will be designed to have the same inclusion and exclusion criteria as this study, and the expected case number will range from 500 to 1000 to raise the power and lower the chance of type I error. The sensitivity, specificity, PPV, and NPV of our SVM prediction model will be calculated and reported. Good performance can be expected. If not, the collection data can be merged with our original training data, giving our model better performance and a lower error rate. It is also possible for researchers from other thalassemia endemic areas to use this same protocol to generate their own thalassemia diagnosis models.

This study has some limitations. First, the number of subjects may not be as large as other SVM prediction model studies [30]. Secondly, there may be heterogeneity among different cohorts/populations and each established machine learning model may need to be modified or re-established when applying to other cohorts. Notwithstanding, our SVM algorithm is applicable and can be extended to other populations.

## 5. Conclusions

SVM is an exceptional technique for distinguishing diseases using simple variables. We developed a convenient tool for primary-care physicians when their deferential diagnoses contain thalassemia for the Taiwanese adult population. This approach, and algorithm, need to be validated in other studies or more extensive databases if possible.

## Figures and Tables

**Table 1:** Demographic characteristics of 350 patients.

	Non-Thalassemia	αT	βT	*p*-Value
Sample size	122 (34.9%)	179 (51.1%)	49 (14%)	---
Age	44.5 (33, 56)	40 (23, 55)	43 (28, 68)	0.118
Sex (female, male)	88, 34	98, 81	28, 21	<0.01
WBC (10^3^/μL)	6055 (4500, 7660)	6020 (5100, 7200)	6200 (4820, 7900)	0.554
RBC (10^6^/μL)	4.58 (4.01, 5.07)	5.43 (4.93, 5.89)	5.1 (4.42, 5.72)	<0.001
Hb (g/dL)	10.85 (8.8, 12.2)	11.8 (10.5, 12.5)	10.9 (9.6, 12)	<0.001
HCT (%)	34.15 (28.3, 38.1)	37.3 (33.7, 40)	33.8 (30.2, 37.4)	<0.001
MCV (fL)	73.8 (65.2, 81.9)	68.5 (65.7, 72)	65.4 (62.6, 75.4)	<0.001
MCH (pg)	23.15 (19.6, 27.1)	21.4 (20.6, 22.4)	21 (20, 24.8)	0.002
MCHC (g/dL)	31.9 (30.3, 33)	31.4 (30.9, 32)	32 (31.4, 32.5)	0.001
RDW (%)	18.35 (15.1, 20.9)	15.6 (14.8, 16.9)	16.3 (15.5, 18.1)	<0.001
Platelet (10^3^/μL)	291 (225,350)	239 (192, 303)	235 (195.5, 281)	<0.001
Mentzer	16.2 (13.3, 19.0)	12.7(11.5, 14.3)	13.7 (10.8, 16.6)	<0.001
RDWI	290.9 (238.9, 353.8)	197.8 (177.1, 239.1)	232.5 (180.3, 305.9)	<0.001
CRUISE	43.62 (42.59, 45.12)	43.1 (42.4, 43.73)	44 (42.94, 44.53)	<0.001
S&L	1241.5 (827.2, 1796.2)	1009.3 (886.9, 1158.6)	895.5 (787.7, 1409.9)	<0.001
Srivastava	5.05 (4.06, 6.3)	3.94 (3.56, 4.47)	4.48 (3.35, 5.18)	<0.001
G&K	97.1 (72.9, 112.1)	63.4 (56.4, 75.4)	70.8 (56.7, 96.3)	<0.001
Sirdah	36.9 (31.1, 42.9)	28.11 (24.2, 33.3)	29.52 (23.0, 37.3)	<0.001
Ehsani	27 (15.5, 39)	14 (8.6, 21.1)	18.3 (4.8, 28.3)	<0.001
E&F	12.3 (6.1, 19.5)	1.4 (−4.1, 8.5)	4.46 (−3.7, 13.9)	<0.001
Ricerca	4.2 (3.0, 4.9)	2.9 (2.6, 3.3)	3.4 (2.8, 4.2)	<0.001
MDHL	1.43 (1.24, 1.6)	1.71 (1.53, 1.84)	1.57 (1.4, 1.83)	<0.001
MCHD	0.32 (0.3, 0.33)	0.31 (0.31, 0.32)	0.32 (0.31, 0.33)	0.001
HH	27.2 (22.7, 32.1)	22.0 (20.7, 24.1)	23.7 (21.4, 28.6)	<0.001

White blood cell (WBC), hemoglobin (Hb), red blood cell (RBC), hematocrit (HCT), mean corpuscular volume (MCV), mean corpuscular hemoglobin (MCH), mean corpuscular hemoglobin concentration (MCHC), red blood cell distribution width (RDW), platelet (PLT), RDW index (RDWI), England and Fraser (E&F), Shine and Lal (S&L), Green and King (G&K), mean density of Hb/liter of blood (MDHL) index, mean cell hemoglobin density (MCHD)Huber–Herklotz index (HH index), alpha-thalassemia (αT), beta-thalassemia (βT).

**Table 2:** Sensitivity, specificity, positive prediction value (PPV), and negative prediction value (NPV) of 13 indices in our study group. *, suggest that the index has the best performance in sensitivity, specificity, PPV, NPV, respectively.

	Sensitivity	Specificity	PPV	NPV
Mentzer index	58.41%	79.51%	84.08%	50.79%
RDWI	62.83%	85.25%	88.75% *	55.32%
CRUISE index	72.12%	27.05%	64.68%	34.38%
Shine and Lal (S&L)	90.27%	37.70%	72.86%	67.65%
Srivastava	41.59%	83.61%	82.46%	43.59%
Green and King (G&K)	55.31%	86.07%	88.03%	50.97%
Sirdah	44.69%	89.34%	88.60%	46.58%
Ehsani	54.87%	77.05%	81.58%	47.96%
England and Fraser (E&F)	42.92%	86.07%	85.09%	44.87%
Ricerca	85.84%	40.16%	72.66%	60.49%
Telmissani- MDHL	40.09%	88.52%	86.67%	44.26%
Telmissani- MCHD	99.12% *	7.38%	66.47%	81.82% *
Huber Herklotz	9.73%	96.72% *	84.62%	36.65%

**Table 3:** The performance comparison for test datasets based on 1000 replications.

Model/Indices	CER (SD)	AUC (95% C.I.)
SVM model	0.26 (0.04)	0.76 (0.69–0.86)
Ricerca	0.68 (0.04)	0.51 (0.40–0.62)
Mentzer	0.44 (0.04)	0.52 (0.39 0.66)
RDWI	0.46 (0.04)	0.51 (0.38 0.64)
CRUISE	0.62 (0.04)	0.57 (0.47 0.67)
MDHL	0.36 (0.04)	0.5 (0.38 0.62)
S & L	0.73 (0.03)	0.48 (0.36 0.59)
Srivastava	0.36 (0.04)	0.52 (0.39 0.65)
G & K	0.41 (0.04)	0.52 (0.39 0.66)
Sirdah	0.36 (0.04)	0.51 (0.39 0.64)
Ehsani	0.43 (0.04)	0.53 (0.40–0.66)
E & F	0.38 (0.04)	0.48 (0.36–0.60)
MCHD	0.82 (0.10)	0.50 (0.47–0.54)

Support vector machine (SVM), classification error rate (CER), area under curve (AUC), confidence interval (C.I.).

## Data Availability

The data presented in this study are available on request from the corresponding author. The data are not publicly available due to institutional restrictions from Taipei Veterans General Hospital.

## Supplementary Materials

The following are available online at https://www.mdpi.com/article/10.3390/diagnostics11091725/s1, Table S1: Formula of 13 indices, Figure S1: Distribution of three groups of patients in 13 indices.
