# Supplementary material for: The TVGH-NYCU Thal-Classifier: Development of a Machine-Learning Classifier for Differentiating Thalassemia and Non-Thalassemia Patients
Source: Diagnostics (Basel). 2021 Sep 20;11(9):1725. doi: 10.3390/diagnostics11091725 (PMC8467438; doi:10.3390/diagnostics11091725)
Supplement: Supplementary file 1 [file diagnostics-11-01725-s001.zip › diagnostics-1333707-SI.pdf]

Table S1. Formula of 13 indices.

Index	Formula	Cut-off point of indicated Thalassemia
Mentzer	MCV/RBC	<13
RDWI	$\frac{MCV \times RDW}{RBC}$	<220
Cruise	$MCHC + 0.603\ RBC + 0.523\ RDW$	>42.63
Shin&Lal (S.L)	$\frac{MCV^2 \times MCH}{100}$	<1530
Sirvastava	MCH/RBC	<3.8
Green & King (G.K)	$\frac{0.01 \times MCV^2 \times RDW}{Hb}$	<65
Sirdah	$MCV - RBC - 3 \times Hb$	<27
Ehsani	$MCH - 10 \times RBC$	<15
England &Fraser (E.F)	$MCV - RBC - 5 \times Hb - 3.4$	<0
Ricerca	RDW/RBC	<44
MDHL	$\frac{RBC \times MCH}{MCV}$	>1.75
MCHD	MCH/MCV	<0.34
Huber-Herklotz (H.H)	$RDW + \frac{0.1 \times MCH \times RDW}{RBC}$	<20

Figure S1. Distribution of three groups of patient in 13 indices.

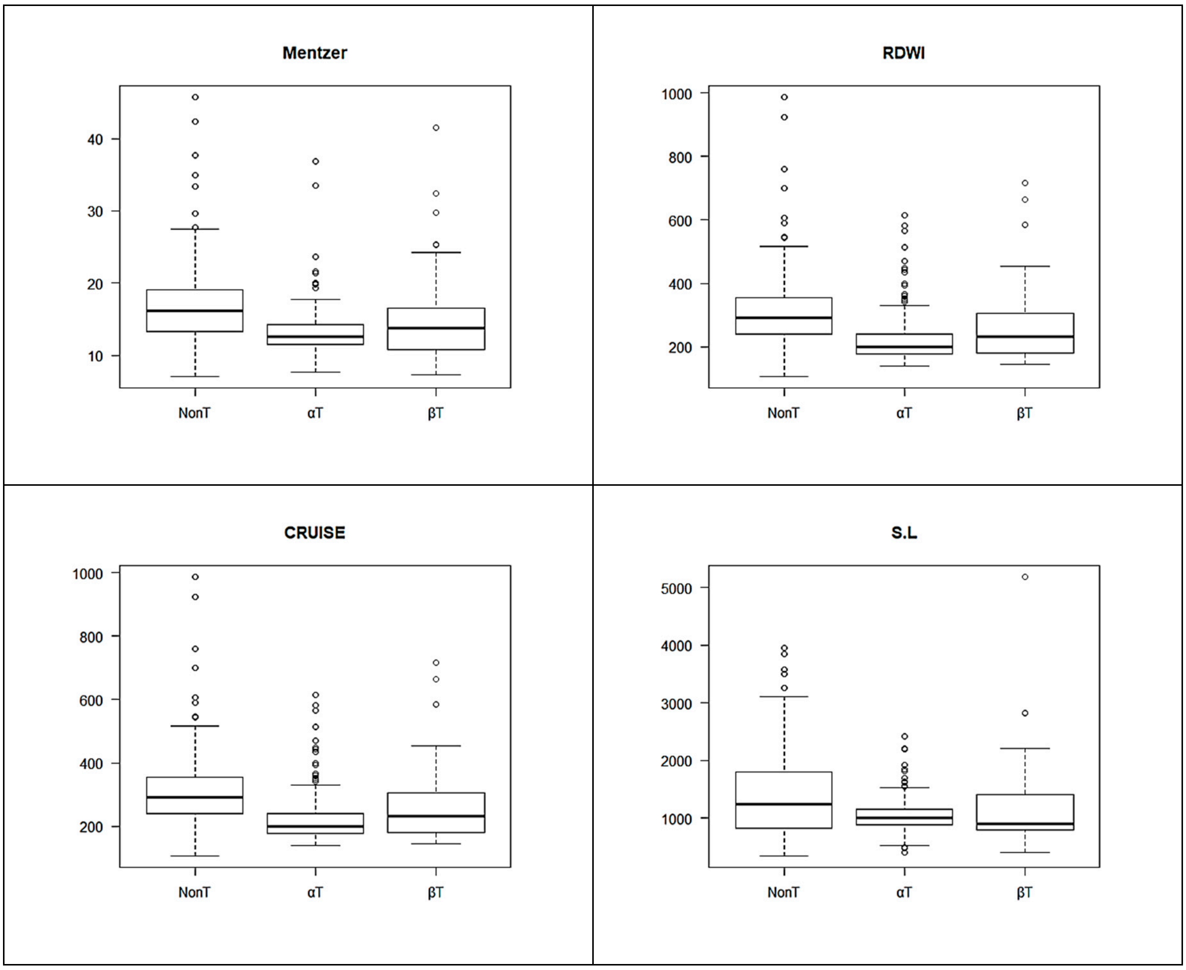

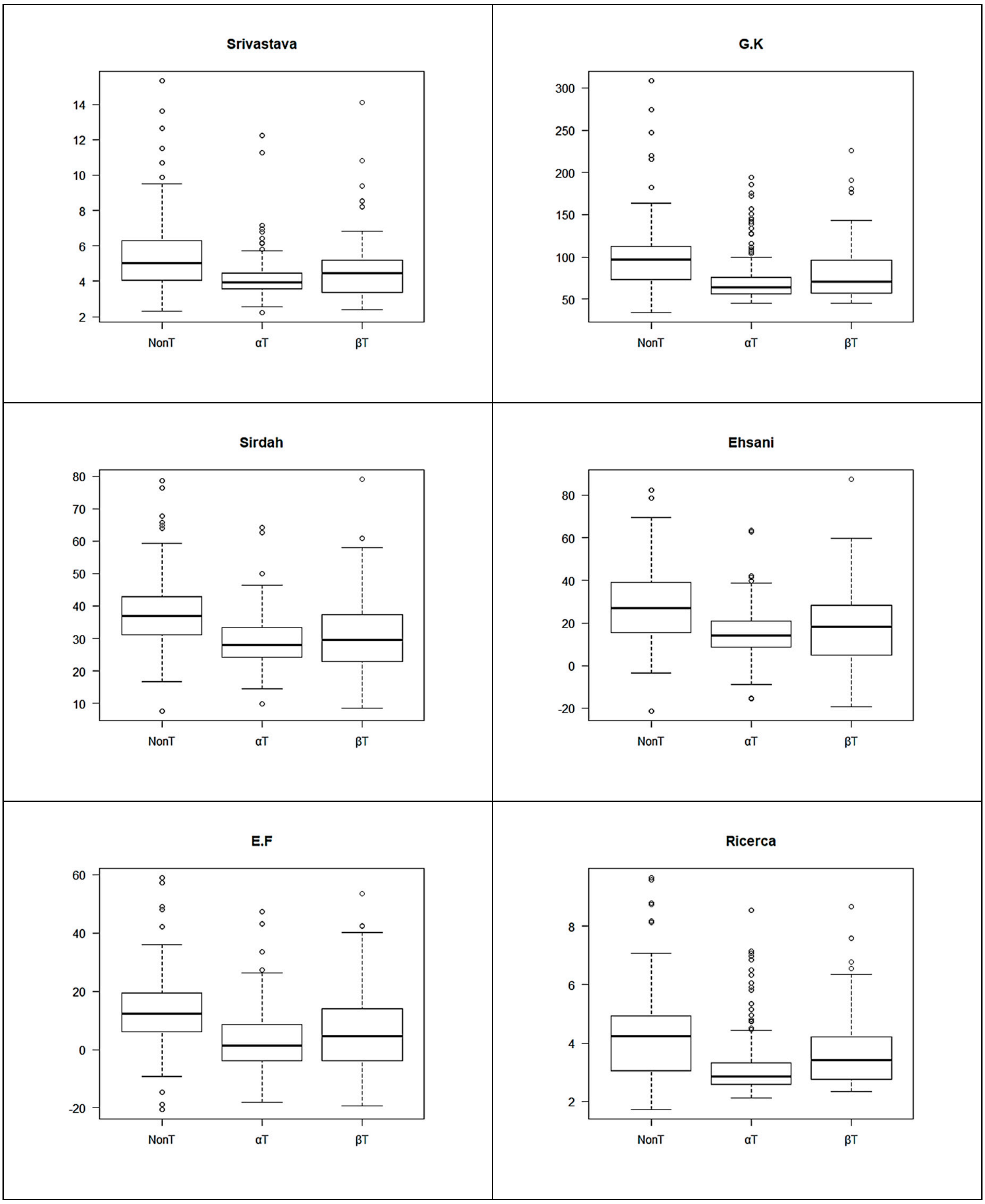
Srivastava
2 4 6 8 10 12 14
NonT αT βT
G.K
50 100 150 200 250 300
NonT αT βT
Sirdah
10 20 30 40 50 60 70 80
NonT αT βT
Ehsani
-20 0 20 40 60 80
NonT αT βT
E.F
-20 0 20 40 60
NonT αT βT
Ricerca
2 4 6 8
NonT αT βT

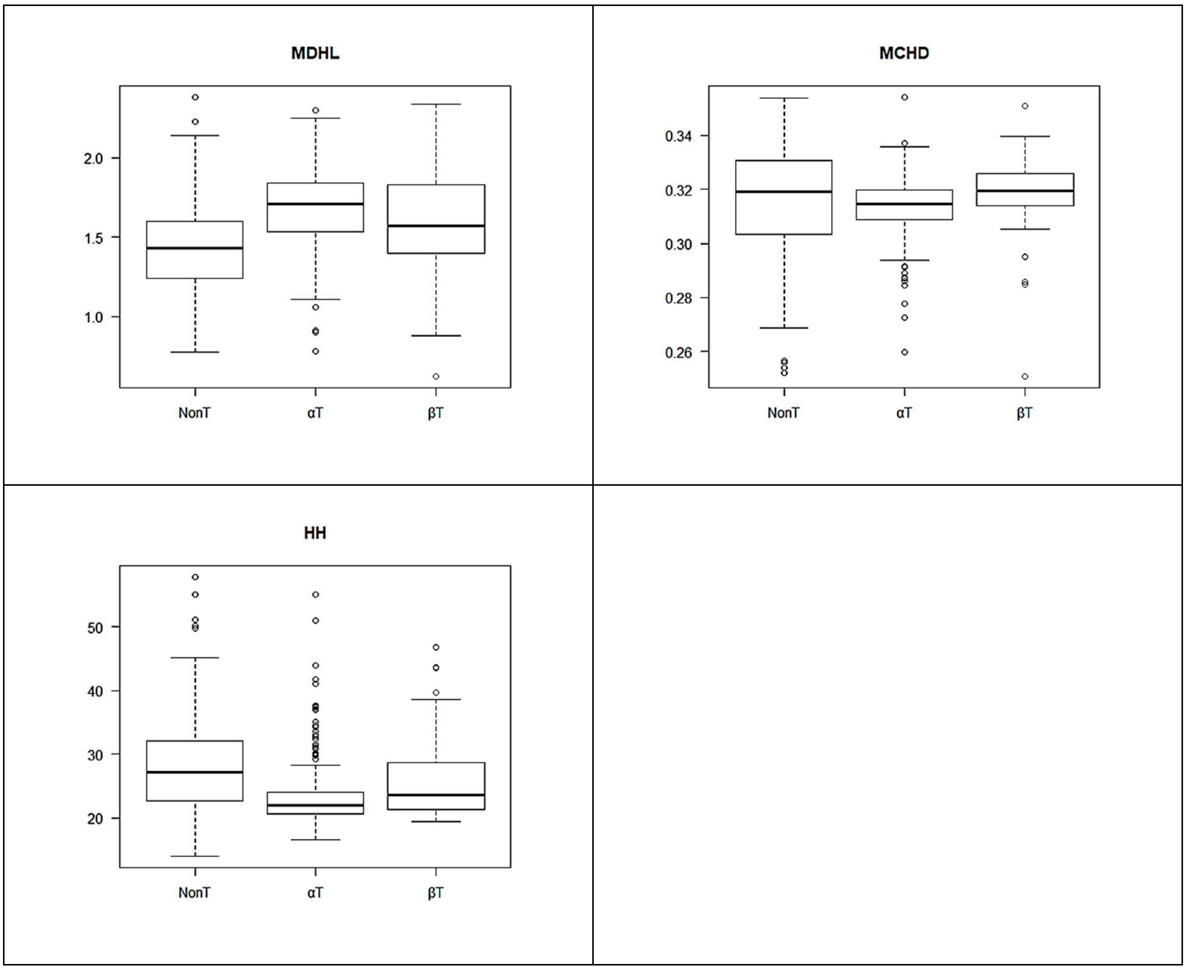
MDHL
2.0
1.5
1.0
NonT
αT
βT
MCHD
0.34
0.32
0.30
0.28
0.26
NonT
αT
βT
HH
50
40
30
20
NonT
αT
βT
